# Diversity Distribution and Assembly Mechanisms of Planktonic and Benthic Microeukaryote Communities in Intertidal Zones of Southeast Fujian, China

**DOI:** 10.3389/fmicb.2019.02640

**Published:** 2019-11-15

**Authors:** Jie Kong, Ying Wang, Alan Warren, Bangqin Huang, Ping Sun

**Affiliations:** ^1^Key Laboratory of the Ministry of Education for Coastal and Wetland Ecosystems, College of the Environment and Ecology, Xiamen University, Xiamen, China; ^2^Fujian Province Key Laboratory for Coastal Ecology and Environmental Studies, Xiamen University, Xiamen, China; ^3^Department of Life Sciences, Natural History Museum, London, United Kingdom; ^4^State Key Laboratory of Marine Environmental Science, Xiamen University, Xiamen, China

**Keywords:** rRNA, diversity, community structure, assembly mechanism, planktonic microeukaryotes, benthic microeukaryotes, intertidal zones, heavy metals

## Abstract

The intertidal zone occupies the shore between the high and low tide marks and is subjected both to natural forces and anthropogenic activities. Compared with the coastal ecosystem, studies comparing diversity and community structure of intertidal planktonic and benthic microeukaryotes are limited. Therefore, the ecological processes mediating their assemblies remain poorly understood. Environmental rRNA from two size fractions (nano- and micro-sized) of plankton and from seasonally collected (spring and summer) benthos, together with water and sediment chemistry and concentrations of heavy metals, were used to explore diversity and community structure of microeukaryotes in intertidal zones of southeast Fujian Province, China. Benthic microeukaryotes harbored significantly higher alpha-diversity than those of the plankton, whereas no distinct patterns of organism size/seasonal distribution were observed for either community. Community compositions differed significantly between planktonic and benthic microeukaryotes, with the former presenting size-fractionated discrepancies and the latter showing seasonal variation. Community turnover between planktonic and benthic microeukaryotes was mainly driven by stramenopiles and alveolates. Distance-decay patterns were found in both communities, with the rate of community turnover being higher for planktonic than benthic microeukaryotes. Among the environmental factors measured, the concentration of Cd and the water content of sediment were closely associated with benthic community variations, whereas none of the factors measured was identified as being responsible for planktonic community variation. Phylogenetic null model analysis indicated that dispersal limitation was the most crucial ecological process mediating community assembly for both planktonic and benthic microeukaryotes in intertidal zones, with heterogeneous selection making a higher contribution to community variation of benthic than planktonic microeukaryotes. Stochastic processes, mainly dispersal limitation, were found to prevail in both communities. This study thus provides new insights into the diversity distribution and assembly mechanism of microeukaryotes in intertidal zones.

## Introduction

Microeukaryotes are extremely diverse organisms that can be found in almost all ecosystems where there is life, playing essential roles in driving biogeochemical cycles and energy flow (Caron et al., 2012; Anderson et al., 2013; De Vargas et al., 2015; Adl et al., 2019). Knowledge of the diversity and patterns of distribution of microeukaryotes, especially their community assembly mechanisms, is therefore of great significance for understanding the ecological processes that maintain ecosystem function and for predicting how ecosystems respond to environmental change at both local and regional scales.

Recently, culture-independent approaches, e.g., high throughput sequencing (HTS), have been developed, which reveal high-yield genetic information with high quality at a fraction of the cost of traditional Sanger sequencing (Goodwin et al., 2016). Such methods enable researchers to comprehensively document microeukaryotes from a variety of marine environments (Gong et al., 2015; Massana et al., 2015; Hindshaw et al., 2017; Shulse et al., 2017; Lentendu et al., 2018). For example, using HTS on the V9 region of the 18S ribosomal RNA gene, De Vargas et al. (2015) investigated the diversity of size-fractionated planktonic eukaryotes in the sunlit ocean, revealing a large number of unassigned OTUs and highlighting the importance of organism size in planktonic community structure. Employing HTS on the V4 region of the 18S ribosomal RNA gene, Forster et al. (2016) found that benthic protists had much higher diversity than planktonic protists in European coastal regions.

Intertidal zones are transitional regions connecting land and seawater. They occupy the shore between the high and low tide marks and therefore are subjected both to natural forces and anthropogenic activities. These ecosystems, therefore, differ significantly from offshore coastal water systems, where conditions are more constant. Recently, several studies have been carried out to investigate microeukaryote diversity and composition in aquatic coastal environments (Logares et al., 2014; Gong et al., 2015; Massana et al., 2015). However, studies regarding intertidal microeukaryotes are still limited, and those dealing with both intertidal planktonic and benthic microeukaryotes are even rarer (Chen et al., 2017; Zhang et al., 2017; Zhu et al., 2017). Chen et al. (2017) showed the intertidal benthic microeukaryotes differ in mechanisms of community assembly compared to coastal planktonic microeukaryotes, with the former exhibiting a significant distance-decay pattern, whereas the latter did not. Zhang et al. (2017) found that the diversity and biogeography of benthic microeukaryotes are driven by environmental factors and dispersal limitation in intertidal sandy beaches, with both making comparable contributions to community variations. Zhu et al. (2017) reported that the community composition of benthic microeukaryotes in an organic carbon-rich mangrove ecosystem was mainly structured by tidal zonation, concentrations of organic matter and sulfates, and sediment grain size. Nevertheless, it is still unclear how spatial and environmental factors affect the diversity and community assembly of planktonic and benthic microeukaryotes in intertidal zones.

Previous studies have demonstrated that variation in rRNA gene copy number among different lineages of microeukaryotes could confuse the results of diversity and community composition, and that rRNA gene transcript-based approaches could mitigate the impact of this (Galluzzi et al., 2004; Not et al., 2009). Also, most investigations on microeukaryotes to date have been based on environmental DNA, which can persist for a long time in a wide variety of marine environments (Dell’Anno and Corinaldesi, 2004), especially sedimentary environments. By contrast, RNA existing in relative active cells is more labile in the environment and measuring this rather than DNA can mitigate the influence of extracellular nucleic acids to some extent, and therefore provide a less biased picture of microeukaryote diversity and community structure (Not et al., 2009). Furthermore, dormant cells could represent a large portion of marine microbial cells, and the rRNA gene transcript-based approach is proposed as a solution for excluding such dormant cells from analyses of microeukaryote (Zinger et al., 2012). This approach has been increasingly used for investigating ocean ecosystems in recent years (Logares et al., 2014; Massana et al., 2015; Xu et al., 2017). However, it is rarely performed to investigate microeukaryotes in intertidal zones.

Understanding the mechanisms that shape and maintain community assembly is central in the study of microbial ecology (Wu et al., 2017; Zhou and Ning, 2017). Deterministic and stochastic processes are two complementary mechanisms in driving microbial assembly (Vellend, 2010; Stegen et al., 2012; Zhou and Ning, 2017). Deterministic processes refer to any selective ecological process, such as environmental factors (e.g., nutrient concentration, salinity, temperature, and pollutant concentration) and biotic interactions (e.g., predation, competition, symbiosis, and parasitism), while stochastic processes refer to any nonselective process, such as ecological drift and dispersal limitation (Zhou and Ning, 2017). Recent progress in sequencing techniques has enabled the documentation of microeukaryote diversity and community composition with high resolution (Logares et al., 2014; Massana et al., 2015; Shulse et al., 2017; Xu et al., 2017), which has motivated a surge of interest in understanding ecological processes governing the assembly and function of microbial communities (Stegen et al., 2012, 2013; Chen et al., 2017; Wu et al., 2017; Zhang et al., 2017). A quantitative understanding of microbial ecology helps to explain the intricate taxonomic pattern observed, predict possible trends of community succession, and may even contribute to manipulating microbial communities toward beneficial states (Xiong et al., 2017; Shi et al., 2018). Previous studies showed that the relative importance of environmental filtering and spatial factors varied among ecosystems, taxa, and regions (Bahram et al., 2016; Chen et al., 2017; Wu et al., 2017). However, mechanisms of assembly of both planktonic and benthic microeukaryotes have rarely been evaluated in intertidal zones.

In the coastal areas of China, thousands of kilometers of intertidal zones are enclosed by seawalls and are facing rapid reclamation for agricultural and industrial uses, potentially turning them into pollutant sinks for inshore and oceanic environments (Ma et al., 2014). Intertidal zones of southeast Fujian, in the western Taiwan Strait, are undergoing rapid urbanization and industrialization and face heavy metal pollution from a range of sources (Yu et al., 2008). Investigating the diversity, community structure, and ecological processes that mediate community assembly of intertidal microeukaryotes can help us to better understand these poorly characterized ecosystems and facilitate the forecasting of possible trends and consequences of community succession.

In the present study, HTS on rRNA gene transcript was used to investigate the diversity, community structure, and shaping factors of both planktonic and benthic microeukaryotes collected simultaneously from the intertidal zone of southeast Fujian province, China. The relative importance of environmental and spatial factors on community variations was quantified using a phylogenetic null model. For planktonic assemblages, pilot studies have shown distinct differentiation among size-fractioned microeukaryotes in marine environments (Logares et al., 2014; De Vargas et al., 2015; Massana et al., 2015). For benthic assemblages of microeukaryote communities in coastal regions, only minor or no seasonal variations were found (Gong et al., 2015; Massana et al., 2015). It is still unknown whether intertidal planktonic and benthic microeukaryotes follow the same pattern as those in coastal water environments. Therefore, the first question explored here is whether the diversity and community composition of intertidal microeukaryotes are related to organism size and seasonal variation. Secondly, we investigate whether planktonic and benthic microeukaryotes occupying distinct ecological niches and playing different ecological functions are likely to have distinct responses to environmental and spatial variations. We hypothesize that intertidal microeukaryotes may be structured by different ecological processes between habitats (i.e., water and sediment).

## Materials and Methods

### Collection of Samples

**Figure 1:**
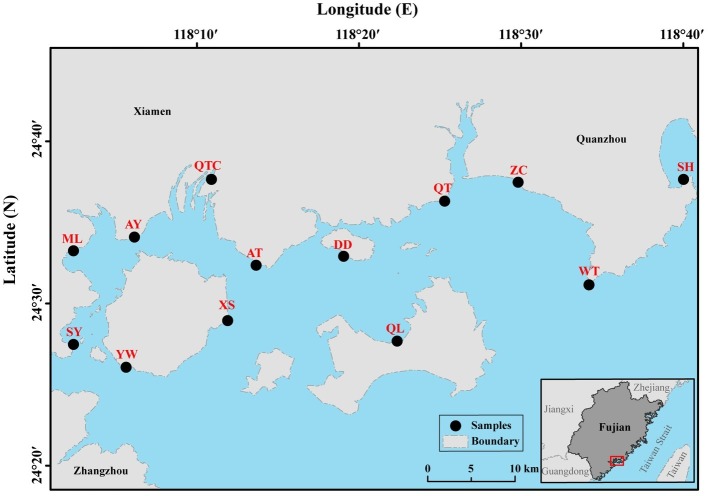
Sampling locations in intertidal zones of southeast Fujian, China. Planktonic and benthic samples were collected from 13 sampling sites (marked by black circles). AT, Ao Tou; AY, Ao Yuan; DD, Da Deng; ML, Ma Luan; QL, Qiong Lin; QT, Qiao Tou; QTC, Qiong Tou Cun; SH, Shen Hu; SY, Song Yu; WT, Wei Tou; XS, Xiang Shan; YW, Yan Wu; ZC, Zhang Cuo.

All 13 sampling sites located in intertidal zones of southeast Fujian, China, are influenced by semidiurnal tidal cycles (Figure 1). The intertidal zones in the study partially located in the tourism city, Xiamen, which is undergoing rapid urbanization and industrialization. Blooms of *Akashiwo sanguinea* (dinoflagellate) frequently occur in spring (February–May) along the shores (Yang et al., 2012). In summer, anthropogenic activity is intense due to large numbers of visiting tourists. Therefore, environmental conditions in the intertidal zone in summer are likely to differ significantly from those in spring, for example, possibly resulting in seasonal difference in the benthos. Altogether, 21 samples for the plankton (10 samples for micro-sized fraction and 11 samples for nano-sized fraction) and 15 for the benthos (8 samples for spring and 7 samples for summer) were collected (Supplementary Table S1). Water (surface layer) was sampled during ebb-tide period in spring of the year 2016 when the water was tens of centimeters’ deep. Sediment (more than 300 g from the top 1 cm) was sampled in both spring (February, 26th–March, 5th; Qiong Lin on April, 27th) and summer (August, 23th–25th) of the same year (Supplementary Table S1).

For the water samples, 2.4 L seawater was collected at each site. The sample was size-fractionated onsite by passing through 20 and 3 μm pore size polycarbonate filter membranes (Millipore, USA). The membrane samples were immersed in RNAlater (Qiagen, Germany) for subsequent RNA analysis. Water salinity and pH were measured onsite using a portable salinity meter (HANNA, China) and benchtop pH meter (Thermo Scientific, USA), respectively. To maximize the number of sampling sites visited during ebb tide and transport the samples to the laboratory as soon as possible, parameters such as concentrations of nutrients and chlorophyll *a* and abundance of bacteria, i.e., that need samples to be fixed onsite, were not measured for plankton. Samples were transported to the laboratory using ice bags to keep them at low temperature and were processed within hours. In the laboratory, membranes immersed in RNAlater were stored at −80°C until RNA extraction. Our preliminary test on intertidal samples showed that it was challenging to get amplicons using the extracted RNA from RNAlater fixed sediments. Therefore, instead of immersing with RNAlater, sediment samples were immediately stored at −80°C after they were transported to the lab. For sediments, duplicate samples were centrifuged at 875 g at 4°C for 5 min, and filtered with 0.22 μm pore-size cellulose acetate membrane to obtain porewater, which was used to analyze salinity, pH, and concentrations of nutrients, i.e., nitrate, nitrite, dissolved silicate, and dissolved reactive phosphorus (Men et al., 2009). Salinity and pH were measured in the same way as for water samples. Concentrations of nutrients were determined using an AA3 Auto-Analyzer (Bran+Luebbe, Germany) (Yan et al., 2012). The water content of the sediment was calculated based on the percentage of weight loss after drying at 60°C for 48 h. The grain size of sediment was measured using a Mastersizer 2000 grain size analyzer (Malvern, UK) according to the manufacturer’s instructions. Chlorophyll *a* (Chl *a*) concentration of sediments was determined by high-performance liquid chromatography after Chl *a* was extracted with N,N-dimethylformamide from sediments freeze-dried at −60°C (Liu et al., 2016). Bacteria were extracted from sediments by treating with tetrasodium pyrophosphate and Tween 80 followed by sonication, stained with 4′,6-diamidino-2-phenylindole (DAPI) and enumerated using epifluorescence microscope at 1,000× magnification under UV excitation (Lei et al., 2010). Metal concentrations were measured using inductively coupled plasma mass spectrometry (ICP-MS, Agilent 7,700×). In brief, around 0.5 g of sediment was microwave-digested in a mixture of concentrated nitric acid (9 ml) and concentrated hydrochloric acid (3 ml) at170°C for 1 h. The digested sediment samples were then centrifuged at 3,000 *g* for 15 min; the supernatant liquid was submitted for metal analysis using ICP-MS (Tan et al., 2013). Metal concentrations in seawater were measured by the diffusive gradients in thin films technique (DGT). A DGT probe was deployed in 25 L of seawater at 25°C for 48 h. Labile metals concentrated by the DGT probe were eluted with 1 mol L^−1^ nitric acid and measured using ICP-MS (Tan et al., 2018).

### RNA Processing and High-Throughput Sequencing

Total RNA extraction and purification were performed using Allprep Total DNA/RNA Mini Kit (Qiagen, Germany) for water samples, and Power Soil Total RNA Isolation Kit (Mo Bio, USA) for sediment samples, following manufacturers’ instructions. RNA extracts were further purified using the DNase kit (Qiagen, German) to remove the remaining DNA. Reverse transcription was performed using the QuantiTect Reverse Transcription Kit (Qiagen, Germany). TAReuk454FWD1 and TAReukREV3 primers were employed to amplify the V4 region of microeukaryotes for both water and sediment samples (Stoeck et al., 2010). The V4 region is the hypervariable region of the 18S rRNA gene, which has been targeted for estimating the environmental diversity of microbial eukaryotes using HTS technologies (Stoeck et al., 2010). Previous studies have shown that the V4 region can yield similar values of diversity and community composition as would have been estimated using the full-length 18S rRNA gene (Dunthorn et al., 2012; Hu et al., 2015). Three PCR products were pooled and purified using the Eastep Gel and PCR Clean-up kit (Promega, China). Sequencing libraries were constructed using TruSeqTM DNA Sample Prep Kit (Illumina, USA) following manufacturers’ instructions. Libraries were sequenced at Majorbio sequencing company (Shanghai, China) using the Illumina MiSeq PE300 platform that covers different variable regions with 2 × 300 bp paired-end reads. All Illumina Miseq raw sequence data were deposited in NCBI Sequence Read Archive (accession code PRJNA549238).

### Data Processing and Statistical Analyses

Raw sequencing data were processed and analyzed using QIIME v.1.8.0 to remove low-quality reads (Caporaso et al., 2010). Chimera removal, singleton removal, and Operational Taxonomic Unit (OTU) clustering were all processed using UPARSE (Edgar, 2013) within the USEARCH v10 package. Representative sequences of each OTU, clustered at 97% similarity cut-off, were blasted against the Protist Ribosomal Reference database (PR2 version 4.7.2) (Guillou et al., 2013). To enable comparison between samples, the OTU table was rarefied to the same sequencing depth of 13,595 reads per sample by randomly resampling, which corresponds to the minimum number of sequences obtained in the samples. Shannon indices were calculated with MOTHUR v.1.25.0 software (Schloss et al., 2009). Faith’s phylogenetic diversity (PD) index (Faith, 1992) was calculated using QIIME.

All statistical analyses were conducted with R software (version 3.3.2) (R Core Team, 2016). Environmental factors, except pH, were subjected to square root transformation, and sequence data were log(x + 1) transformed before downstream analyses. Principal component analysis (PCA) was used to reveal the distribution of environmental parameters. Spearman’s rank correlation coefficients were calculated to explore the relationship between alpha diversity and environmental factors with *p*-values corrected using the “holm” method in R. Non-metric multidimensional scaling analysis (NMDS) based on Bray-Curtis dissimilarity matrix was used to detect possible community patterns which were further assessed using analysis of similarity (ANOSIM) (Oksanen et al., 2016). Mantel and Partial Mantel tests were performed to reveal the potential factors correlating with community variations. To test whether there was a distance-decay relationship, Spearman’s rank correlation coefficients between community similarity matrix and geographic distance was calculated. To identify OTUs contributing the most to differences between groups under each condition, i.e., habitats, organismal sizes and seasons, package edgeR was used to reveal the relatively abundant OTUs with sequences making up more than 0.5% of total sequences (Robinson et al., 2010). A phylogenetic null model was applied using an approach based on phylogenetic and taxonomic diversities in order to quantify microeukaryotic community assembly processes, such as selection, dispersal limitation, ecological drift, and homogenizing dispersal (Stegen et al., 2013). To quantify ecological processes mediating the community assembly of microeukaryotes, two major steps were processed. First, the β-nearest taxon index (βNTI) for all pairwise community comparisons was calculated by quantifying the magnitude of deviation between the observed degree of phylogenetic turnover and null distribution of phylogenetic turnover. Second, Bray-Curtis-based Raup-Crick (RC_bray_) for pairwise community comparisons were evaluated by characterizing the magnitude of deviation between observed OTU composition turnover and null distribution of OTU composition turnover (Stegen et al., 2013).

## Results

### Environment of Sampling Sites

The distribution of environmental parameters was revealed by principal component analysis (PCA) (Supplementary Figure S1). In general, the dynamics of environmental factors from sedimentary samples were more complex than those of water samples. Salinity, pH, and concentrations of Cd, Zn, Cu, and Ni in water samples were all statistically lower than in sedimentary samples (*t*-test, *p* < 0.05) (Supplementary Figure S2). However, environmental factors of sediment showed insignificant seasonal variations (ANOSIM, *R* = 0.109, *p* = 0.122). Comparison of the individual environmental parameter between seasons showed similar results (*t*-test, *p* > 0.05), i.e., As (*p* = 0.34), Cd (*p* = 0.22), Cu (*p* = 0.43), Ni (*p* = 0.71), and Zn (*p* = 0.25).

### Alpha Diversity of Planktonic and Benthic Microeukaryotes

**Figure 2:**
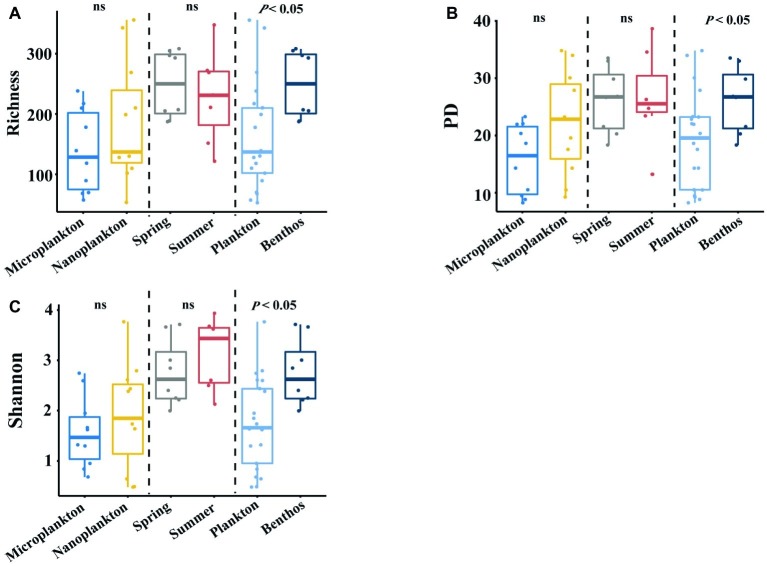
Comparing α-diversity of intertidal microeukaryotes between or among different groups. **(A–C)** boxplots show Richness, PD, and Shannon values among different comparisons, with a vertical dotted line dividing into three parts, which are based on different grouping standards. For the comparison between plankton and benthos, only samples from the spring season are considered. Pairwise comparisons are tested using one-way ANOVA. Ns means that *p* is not significant. Dashed lines denote the separation of each condition, i.e., habitats, organismal sizes, and seasons.

Planktonic (21 samples) and benthic (15 samples) datasets yielded 1,051,532 and 299,961 clean amplicons in total, respectively. With a cutoff value of 97% similarity, 1,046 and 846 OTUs were recovered for planktonic and benthic microeukaryotes, respectively, after removing singletons and multicellular eukaryotes (metazoa and plantae). The alpha-diversity of intertidal microeukaryotes differed significantly between habitats, with benthic microeukaryotes harboring significantly higher diversity (on average 249 OTUs) than their planktonic counterparts (on average 163 OTUs). This distribution pattern was consistent among diversity indices (phylogenetic diversity, 26.2 ± 2.0 in sediments vs. 19.1 ± 8.2 in seawater; Shannon, 2.76 ± 0.23 in sediments vs. 1.74 ± 0.89 in seawater) (Figures 2A–C). For the comparison between plankton and benthos, only samples from the spring season are considered. However, no statistical difference was found between organism sizes and seasons (Figures 2A–C).

Planktonic and benthic microeukaryotes showed an inconsistent pattern in correlations with environmental parameters (Supplementary Tables S2, S3). Alpha diversity of planktonic microeukaryotes failed to correlate with any environmental parameter measured, whereas that of benthic microeukaryotes showed a significant correlation with concentrations of Cd, Cu, and NO_x_, and grain sizes (Supplementary Tables S2, S3). In planktonic microeukaryotes, the alpha-diversity of microplankton negatively correlated with concentrations of Ni and Mn (*r* values in a range of 0.700–0.733, *p* < 0.05), while that of nanoplankton showed a negative correlation with concentration of Se (*r* = −0.636, *p* < 0.05; Supplementary Table S2). In benthic microeukaryotes, the alpha-diversity of spring communities showed correlations with salinity, concentration of Pb, and grain size, whereas that of summer communities significantly correlated with pH, concentrations of Cd, Cu, Zn, phosphate, water content and grain size (Supplementary Table S3). The alpha-diversity of intertidal microeukaryotes exhibited a distinct correlation pattern with environmental factors between habitats (water and sediment), organism body sizes (nano- and microplankton) and seasons (spring and summer).

### Community Composition of Planktonic and Benthic Microeukaryotes

**Figure 3:**
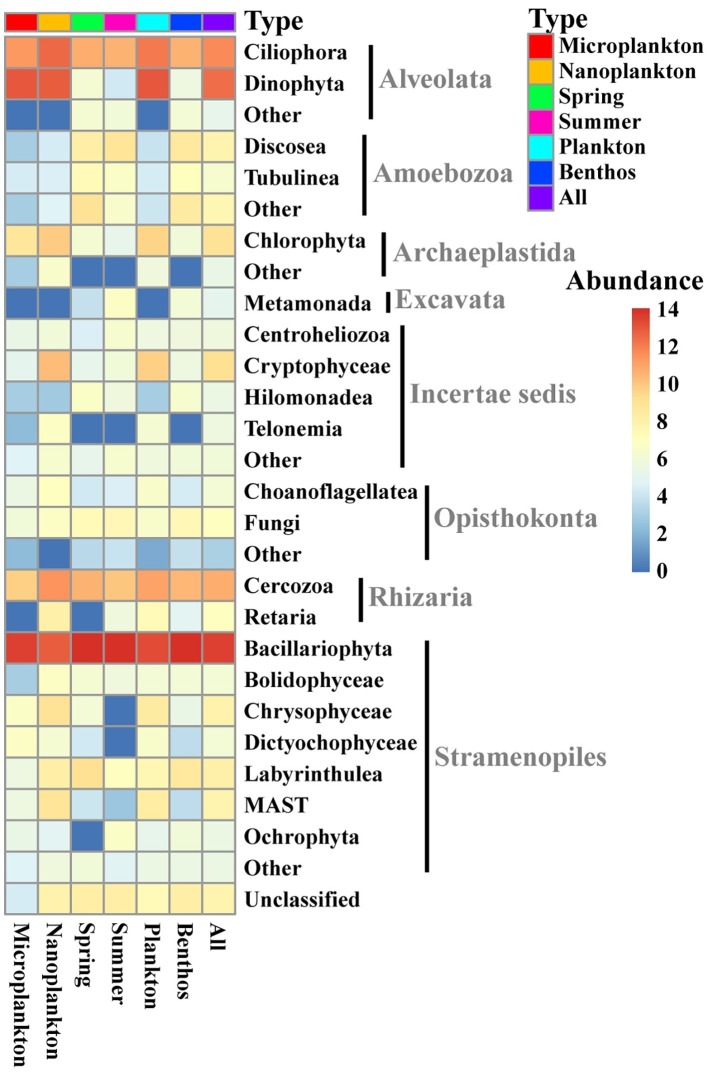
Heatmap showing the distribution and composition of intertidal microeukaryotes. The relative sequence abundances assigned to the 20 most abundant groups of all intertidal microeukaryotes are shown with the rest merged into “Other.” The sequence data were rarefied to 13,959 and log-transformed for each sample and averaged in different groups. Top row shows types of sample marked with different colors. Microplankton and Nanoplankton refer to size-fractionated water samples collected in spring; Spring and Summer represent sediment sampled in spring and summer, respectively; Plankton includes Microplankton and Nanoplankton, while Benthos includes samples collected in both Spring and Summer. All represents all samples.

**Figure 4:**
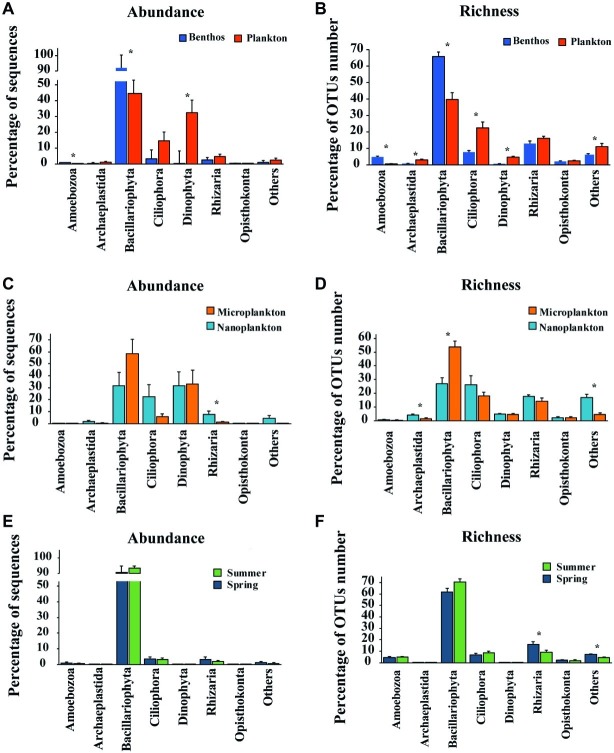
Comparisons of relative abundance of sequence and OTUs number (richness) of major groups between Plankton **(A)** and Benthos **(B)**; between Microplankton **(C)** and Nanoplankton **(D)**; between Spring **(E)** and Summer **(F)**. The relative abundance of any given group is the proportion of the sequences of that group to the total number of sequences in the sample, while the relative OTUs number (richness) is the proportion of OTUs assigned to any given group to the total OTUs number of OTUs in the sample. Asterisks denote significant differences (one-way ANOVA, *p* < 0.05). Microplankton and Nanoplankton refer to size-fractionated water samples collected in spring; Spring and Summer represent sediment sampled in spring and summer, respectively; Plankton includes Microplankton and Nanoplankton, while Benthos includes samples collected in both Spring and Summer.

All sequences were assigned to 28 lineages within seven recognized supergroups, namely Alveolata, Amoebozoa, Archaeplastida, Excavata, Opisthokonta, Rhizaria, and Stramenopiles (Figure 3). Stramenopiles dominated the total microeukaryote community in terms of numbers both of sequences and OTUs (average proportion of total sequence abundance and OTU richness about 65.1 and 57.8%, respectively), followed by Alveolata (average proportion of total sequence abundance and OTU richness about 28.9 and 15.7%, respectively) and Rhizaria (average proportion of total sequence abundance and OTU richness about 3.8 and 15.6%, respectively) (Figures 3, 4A,B).

For planktonic communities, the stramenopiles group Bacillariophyta (average abundance about 44.6 ± 8.4%) and the alveolate group Dinophyta (average abundance about 32.4 ± 7.9%) were the major contributors of sequence abundance, whereas benthic communities were dominated by Bacillariophyta alone (average abundance about 92.0 ± 2.2%) (Figure 4A). Size-fraction subcommunities of planktonic microeukaryotes (i.e., microplankton and nanoplankton), and seasonal subcommunities of benthic microeukaryotes (i.e., spring and summer) showed similar trends to that of the total community (Figures 4C,E). For OTU richness, the major contributors of planktonic and benthic communities were Bacillariophyta (39.7 ± 4.2 and 65.8 ± 2.7%, respectively), Ciliophora (22.5 ± 3.6 and 7.7 ± 1.0%, respectively), and Rhizaria (16.1 ± 1.2 and 12.9 ± 1.6%, respectively) (Figure 4B). Similarly, subcommunities of microplankton and nanoplankton and of benthic community in spring and summer showed a similar trend (Figures 4D,F).

Statistical analysis showed that 27 OTUs contributed the most to differences between plankton and benthos as determined by edgeR (Supplementary Figure S3A). About 12 of the 27 OTUs enriched in sediment were assigned to Bacillariophyta (e.g., OTU14, OTU89, OTU44, OTU15, and OTU27), while the rest 15 OTUs enriched in water that were assigned to Ciliophora (e.g., OTU7, OTU5, OTU45, and OTU36), Dinophyta (OTU1), Rhizaria (e.g., OTU42, OTU18, and OTU19), and Bacillariophyta (e.g., OTU23, OTU4, and OTU17). For planktons, six OTUs identified using edgeR enriched in microplankton that was assigned to Bacillariophyta, while eight OTUs enriched in nanoplankton that was assigned to Ciliophora, Rhizaria, Bacillariophyta, and Cryptophyta (Supplementary Figure S3B). The most 12 differential OTUs between benthos sampled in spring and summer were all assigned to Bacillariophyta, with 5 of the OTUs enriched in the samples collected in spring (Supplementary Figure S3C).

### Community Structure of Plankton and Benthos and Their Shaping Factors and Assembly Mechanisms

**Figure 5:**
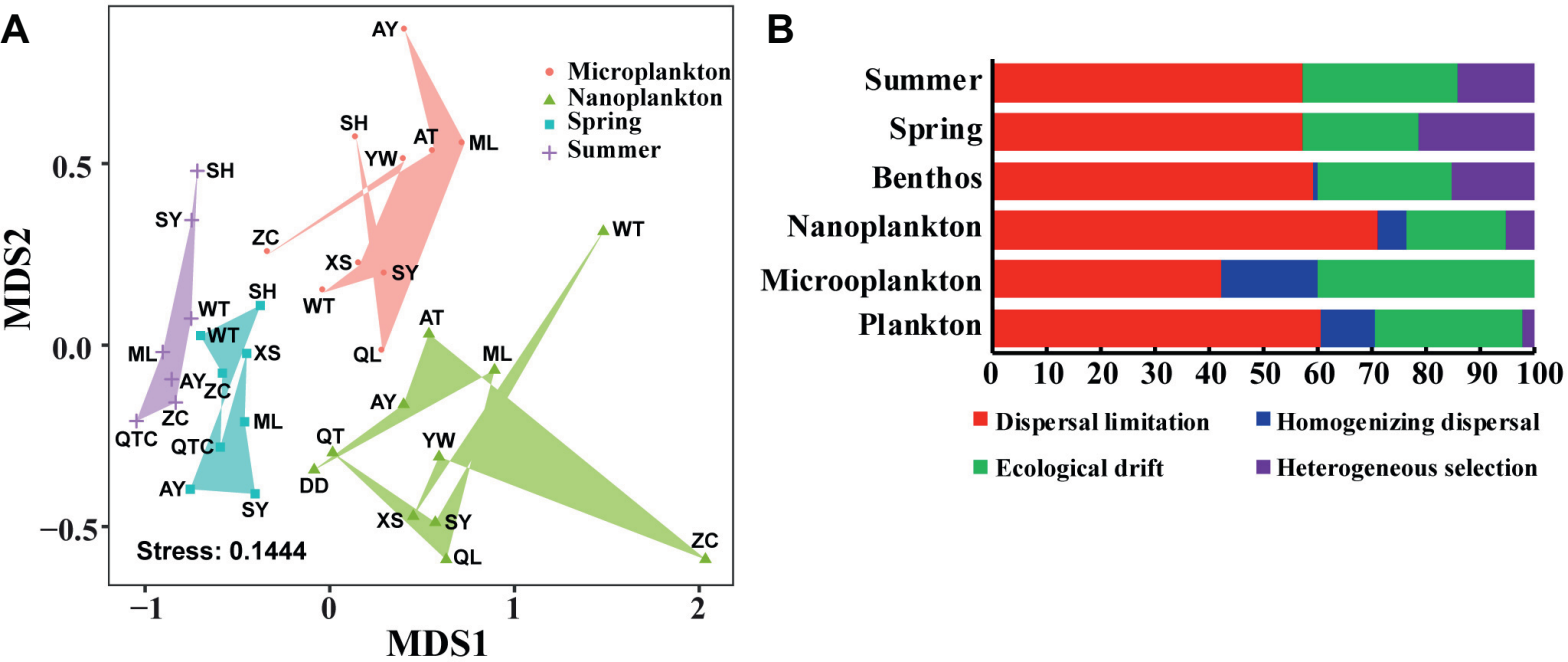
Distribution pattern and assembly mechanism of microeukaryotes in intertidal zones. Non-metric multidimensional scaling (NMDS) of intertidal microeukaryote communities based on Bray-Curtis distance **(A)**. Quantification of ecological processes shaping microeukaryotes in intertidal zones **(B)**. Microplankton and Nanoplankton represent size-fractionated water samples, while Spring and Summer represent sediment sampled in spring and summer, respectively; Plankton includes Microplankton and Nanoplankton, while Benthos includes samples collected in both Spring and Summer.

Planktonic and benthic microeukaryote communities clustered into two groups, and each exhibited distinct patterns, with benthic communities clustering into spring and summer subgroups and planktonic communities grouping into nano- and micro-size subgroups (Figure 5A). These clusters were verified by ANOSIM with an overall *R* of 0.584 and pairwise *R* ranging from 0.239 to 0.734, indicating significant differences in community composition between groups compared (Table 1).

**Table 1:** Analysis of similarities (ANOSIM) of subcommunities or whole communities by habitat, organismal size, and season.

Grouping	*R*	*P*
Plankton vs. benthos	0.660	<0.001
Microplankton vs. nanoplankton	0.299	<0.001
Spring vs. summer	0.445	<0.001
All groups	0.584	<0.001

*Plankton and benthos refer to all 21 water and 15 sediment samples; Microplankton (10 samples) and Nanoplankton (11 samples) refer to size-fractionated water samples collected in spring; Spring (8 samples) and Summer (7 samples) refer to sediments sampled in spring and summer, respectively. The ANOSIM is based on Bray-Curtis similarity (permutations = 9,999)*.

**Figure 6:**
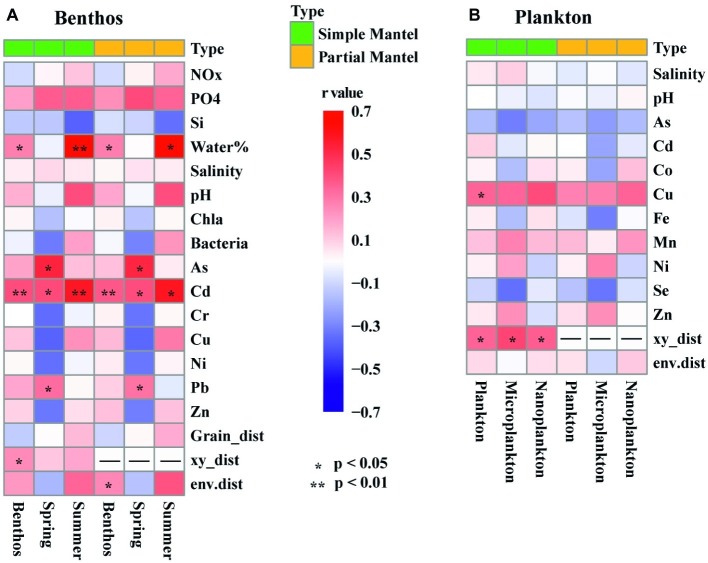
Heatmaps showing the results of simple and partial Mantel tests for the correlations between environmental factors, spatial variables, and intertidal benthic **(A)** and planktonic **(B)** microeukaryotes. The colored boxes represent *r* values, asterisks denote significant *p* values (< 0.05). Benthos represent all 15 benthic samples. Spring (8 samples) and Summer (7 samples) represent sediment sampled in spring and summer, respectively. Plankton represents all 21 planktonic samples collected in spring. Microplankton (10 samples) and Nanoplankton (11 samples) refer to size-fractionated water samples collected in spring. Community variations were based on Bray-Curtis similarity. Significances tests are set at 9999 permutations, and *p* <0.05 and <0.01 are marked with * and **, respectively. Abbreviations: NOx, nitrate, and nitrite; PO4, dissolved reactive phosphorus; Si, dissolved silicate; Water%, water content of the sediment. As, arsenic; Cd, cadmium; Co, cobalt; Cr, chrome; Cu, copper; Fe, iron; Mn, manganese; Ni, nickel; Pb, lead; Se, Selenium; Zn, zinc; Grain_dist, Euclidean distance of sediment grain size; xy_dist, pairwise geographical distances based on Cartesian coordinates generated from longitude and latitude; env.dist, Euclidean distance of all environmental factors.

Among the 22 measured environmental factors, the concentration of Cu and geographic distance were found to be important influencing factors on the planktonic community, whereas the concentration of Cd, the water content of sediment, and geographic distance significantly correlated with community variation of benthic microeukaryotes (Figure 6). Negative relationships between community similarity and geographic distance were found in both planktonic and benthic microeukaryote communities, with the former exhibiting a relatively stronger distance-decay pattern (*r* = −0.383, *p* < 0.001) than the latter (*r* = −0.264, *p* = 0.007) (Supplementary Figure S4).

The phylogenetic null model analysis showed that dispersal limitation prevailed in both planktonic and benthic communities under each condition, i.e., between organism body sizes and seasons, with homogenizing dispersal exhibiting a stronger effect on community assembly of the plankton than the benthos whereas the reverse was true for heterogeneous selection (Figure 5B). In planktonic microeukaryotes, dispersal limitation had a larger effect on nanoplankton (70.9%) than microplankton (42.2%) whereas homogenizing dispersal (17.8 vs. 5.5%) and ecological drift had a greater effect on microplankton than nanoplankton (40.0 vs. 18.2%) (Figure 5B). In benthic microeukaryotes, dispersal limitation (57.1 vs. 57.1%) dominated in both spring and summer communities, followed by ecological drift (21.4 vs. 28.6%) and heterogeneous selection (21.4 vs. 14.3%), with homogenizing dispersal making an extremely low contribution to community variation in different seasons (Figure 5B).

## Discussion

In the present study, no replicates for each sampling site were collected; therefore, it is impossible to evaluate the variation within each location. The following discussion does not take into account intra-site variations.

### Alpha and Beta Diversity and Influencing Factors

Our results showed that sediment harbored a higher diversity of microbial eukaryotes than water in intertidal zones (Figures 2A–C). A similar pattern was observed in coastal regions of Europe (Massana et al., 2015; Forster et al., 2016). This has been attributed to physicochemical microhabitat heterogeneity, niche partitioning, and allopatric speciation (Forster et al., 2016). Therefore, the most plausible reason for the alpha distribution pattern seen in intertidal zones was the higher microhabitat heterogeneity of intertidal sediments compared to the overlying water (Supplementary Figure S2). This heterogeneity is probably induced by a combination of daily tides, waves, and anthropogenic activities, which create a wider range of ecological niches compared to water, which is a relatively homogeneous environment because it is well mixed and subject to long-distance transportation by tides and winds.

By employing high throughput sequencing on the rRNA gene, previous studies on microeukaryotes showed that alpha diversity was about one order of magnitude higher than that in the present study in the same region (Chen et al., 2017; Zhang et al., 2017). We speculate that the difference might be due to any or all of the following reasons. First, data generated from different sources might contribute to the apparent difference in alpha diversity. Environmental RNA, used in the present study, is thought to exist in living, ribosomally active cells only (Blazewicz et al., 2013), whereas environmental DNA is known to have long persistence times in marine environments (Dell’Anno and Corinaldesi, 2004). Secondly, the target region chosen as a marker gene to explore alpha diversity might explain part of the variance. A recent study has shown that the V4 region (used in this study) could result in 20% less OTUs than the V9 region (used in previous studies) (Tragin et al., 2018). Thirdly, there were inconsistencies in sampling strategies. In the present study, total RNA was extracted from sediments directly without any pre-processing in order to minimize the loss of microorganisms. Chen et al. (2017) and Zhang et al. (2017) sampled benthic microeukaryotes by filtering samples onto polycarbonate membranes after mechanically shaking and separating microeukaryotes from the sand. Therefore, to make results from different investigations comparable, standard procedures for sampling, sample processing and data analyses should be established.

Interestingly, this study showed a much higher percentage of shared OTUs between planktonic and benthic microeukaryotes (56.39 and 60.99%, respectively) than previous studies on microeukaryotes in coastal regions (Supplementary Figure S5). For example, Chen et al. (2017) found that 18.53% of total OTUs were shared by both groups in Xiamen Bay, Fujian Province, China. In a coastal region of Europe, Forster et al. (2016) reported that 12.89 and 11.48% of OTUs were shared by water (i.e., surface and Deep Chlorophyll Maximum layers) and sediment, respectively. Compared with coastal regions, however, intertidal zones are much more dynamic because of strong perturbation by cyclical fluctuations resulting in stronger mixing between water and sediment. This mixing might enhance the exchange between planktonic and benthic microeukaryote communities, producing a high percentage of shared OTUs by the two groups in intertidal zones. Also, sediment serving as a sink could accumulate microeukaryotic cells from the water above, which could result in more shared OTUs between plankton and benthos. Finally, methodological differences might contribute to the discrepancies among these studies.

Correlation analyses showed that none of the environmental factors measured was correlated with alpha diversity of planktonic microeukaryotes, whereas benthic microeukaryotes were negatively correlated with concentrations of Cd, Cu, NO_x_, and grain size (Supplementary Tables S2, S3). Statistical analyses showed that the environmental heterogeneity of sediment was significantly higher than that of water (Supplementary Figure S2), with salinity, pH, and concentrations of Zn, Cd, Cu, and Ni significantly higher in sediment than water (*t*-test, all *p* < 0.05). Therefore, the distinct pattern of correlational relationships between planktonic/benthic microeukaryotes and environmental factors might be due to sediment exerting a stronger selection than water as a result of the significantly higher salinity, pH, and concentrations of heavy metals in the benthos.

Previous studies have shown that there are no consistent effects of heavy metals on prokaryote or microeukaryote diversity. For example, Gong et al. (2015) reported that in the Yellow Sea, alpha diversity of planktonic microeukaryotes is positively correlated with concentrations of Mn and As while that of benthic microeukaryotes was negatively correlated with the concentration of Zn. The diversity of bacteria in sediments in an estuary of southeast Australia and in a coastal region of Italy was found to be negatively correlated with heavy metals (Sun et al., 2012; Quero et al., 2015). However, no apparent relationship between bacterial diversity and heavy metals was found in sediments in the Xiangjiang River, China (Zhu et al., 2013). High concentrations of heavy metal may not necessarily be toxic because bioavailability and various physicochemical factors can strongly influence levels of biotoxicity. Furthermore, Guo et al. (2017) found that sensitivity and tolerance of soil bacteria to heavy metals differed among microbial groups. Therefore, the effects of heavy metals on microbial diversity, both prokaryote, and eukaryote, remain unclear.

### Community Structure of Intertidal Microeukaryotes

Stramenopiles (mainly Bacillariophyta) and Alveolata (mainly Dinophyta and Ciliophora) dominated both the planktonic and benthic microeukaryote communities in the intertidal zone in terms of sequence abundance and OTU richness (Figures 3, 4). This is consistent with the findings of previous studies in other coastal regions (Gong et al., 2015; Massana et al., 2015; Chen et al., 2017; Zhang et al., 2017).

Bacillariophyta, serving as primary producers, was the dominant group, although its composition differed significantly in the planktonic vs. benthic communities (ANOSIM, *R* = 0.5389, *p* = 0.001; Figures 4A,B; Supplementary Figure S3A). Nevertheless, a significant fraction of Bacillariophyta OTUs (57.3% of all Bacillariophyta OTUs in whole intertidal community) was shared by the two groups and accounted for 87.0% of planktonic and 63.6% of benthic Bacillariophyta OTUs, respectively. This indicates a high percentage of exchange between planktonic and benthic communities due to the strong mixing of water and sediment, probably by the action of daily tides and winds. This might imply the essential roles of sediment in receiving and accumulating microeukaryotes from the water above.

Based on relative sequence abundance, Dinophyta was the second largest group of intertidal planktonic microeukaryotes (Figure 4A). This contrasts with the findings of studies in European coastal and Yellow Sea coastal waters where the relative abundance of Dinophyta was higher than that of Bacillariophyta (Gong et al., 2015; Massana et al., 2015). This contrasting result might be ascribed to the different types of nucleic acids used (rRNA vs. rDNA) and differences between the ecosystems investigated. For example, Not et al. (2009) found that the relative contribution of Dinophyta to the whole microeukaryote community was higher when this was analyzed using rDNA vs. rRNA. This finding might be related to the large rDNA copy number in dinophytes and the persistence of extracellular DNA in the environment (Dell’Anno and Corinaldesi, 2004; Galluzzi et al., 2004). It is also noteworthy that the relative sequence abundance of Dinophyta differed significantly between water and sediment (Figure 4A; Supplementary Figure S3A), contributing only about 0.02% to the benthic microeukaryote community (Figure 4A). Furthermore, four out of six Dinophyta OTUs recovered from benthic communities were shared with planktonic communities, indicating that most Dinophyta found in sediment might have originated from water above. However, rRNA gene transcript-based amplification was applied in the present study to mitigate the impact of the presence of dormant cells. Combined with the difficulty in extracting nucleotides from dormant cells, the presence of dinoflagellates in sediments could, therefore, be underestimated.

Intertidal planktonic and benthic microeukaryotes showed significant differences in community composition, with planktonic microeukaryotes having organismal size fraction differences and benthic microeukaryotes exhibiting seasonal variations (Figures 3–5A; Table 1). Significant differences between planktonic and benthic communities have been observed in both prokaryotes and microeukaryotes in a wide variety of environments (Zinger et al., 2011; Massana et al., 2015; Chen et al., 2017; Shulse et al., 2017). For planktonic assemblages, pilot studies have shown distinct differentiation among size-fractioned microeukaryotes in marine environments (Logares et al., 2014; De Vargas et al., 2015; Massana et al., 2015). In contrast, Gong et al. (2015) found only minor seasonal variations in benthic microeukaryote communities in coastal regions of the Yellow Sea. Compared with the offshore coastal environment, sediment characteristics of intertidal zones could exhibit a higher fluctuation because of daily tidal cycles and seasonal variations, resulting in a more dynamic benthic microeukaryote community composition.

### Factors and Ecological Processes Controlling Community Assembly

Partial Mantel tests showed that environmental factors, including the concentration of Cd, the water content of sediment, and geographic distance, were significantly correlated with benthic microeukaryotes, whereas only geographic distance was correlated with planktonic microeukaryotes (Figure 6). This suggests that different forcing factors shaped the community structure of planktonic vs. benthic microeukaryotes in intertidal zones. Chen et al. (2017) reported similar findings in an investigation on patterns and processes in microeukaryotes from coastal waters and intertidal sediments of Xiamen Island, Fujian Province, China. In that study, it was concluded that environmental and spatial factors were responsible for community variations of benthic microeukaryotes whereas neither environmental nor spatial factors were correlated with those of planktonic microeukaryotes (Chen et al., 2017). When comparing the effects of environmental factors measured in the present study, it was found that the heterogeneity of the environment was significantly higher in sediment than in water (*t*-test, *p* < 0.001). The homogeneity of the water environment might explain why there was no apparent relationship between environmental factors and community variation. On the other hand, fewer environmental factors were measured for planktonic samples (12 vs. 17 in benthic samples), which could be another possible reason for this difference. A previous study showed that despite a relatively large number of environmental factors measured, the proportion of community variations explained by the environment remains low (Zhou et al., 2008). This indicates that many unmeasured but essential factors and processes, such as dispersal, ecological drift, water currents, and biotic interactions (e.g., predation and competition), could play important roles in shaping community assemblies (Nemergut et al., 2013; Bahram et al., 2016; Chen et al., 2017; Wu et al., 2017).

A significant distance-decay pattern was found in both planktonic and benthic microeukaryotes in intertidal zones in this study. By contrast, Chen et al. (2017) found that spatial effects occurred in intertidal benthic microeukaryotes but were absent in coastal planktonic microeukaryotes. We collected samples from intertidal zones along a shoreline across 66 km in southeast Fujian, China (Figure 1), whereas Chen et al. (2017) collected samples from Xiamen Island that covered a relatively smaller area. Studies on the distance-decay patterns of soil bacteria at multiple spatial scales showed that homogenizing dispersal dominates at small spatial scales leading to a large community similarity at closely located sites (Feng et al., 2019). Therefore, the inconsistency of distance-decay patterns in planktonic microeukaryotes in the two studies may be attributed to the difference in sampling scales (e.g., 6.04–66.48 km in the present study vs. 1.69–19.96 km around Xiamen Island).

Understanding the mechanisms that shape and maintain community assembly is central to the study of microbial ecology (Wu et al., 2017; Zhou and Ning, 2017). Deterministic and stochastic processes are two complementary mechanisms that drive the assembly of microbial communities (Vellend, 2010; Stegen et al., 2012; Zhou and Ning, 2017). Deterministic processes refer to any selective ecology processes, such as environmental factors (e.g., nutrient concentration, salinity, temperature, and pollutant concentration) and biotic interactions (e.g., predation, competition, symbiosis, and parasitism), while stochastic processes refer to any nonselective processes, such as ecological drift and dispersal limitation (Zhou and Ning, 2017). Although some processes, e.g., biotic interactions, are challenging to quantify, deterministic and stochastic processes can still be investigated by calculating the relative importance of environmental filtering and spatial factors in shaping natural community assembly (Stegen et al., 2012; Zhou and Ning, 2017). Previous studies showed that the relative importance of environmental filtering and spatial factors varied among ecosystems, taxa, and regions (Bahram et al., 2016; Chen et al., 2017; Wu et al., 2017). Hitherto, no studies have simultaneously investigated assembly mechanisms of planktonic and benthic microeukaryote communities in intertidal zones. To evaluate the potential effects of deterministic and stochastic processes on planktonic and benthic microeukaryotes in intertidal zones, phylogenetic null model analyses were performed (Sloan et al., 2006; Logares et al., 2013; Chen et al., 2017). It was found that dispersal limitation and ecological drift were dominant ecological processes for both planktonic and benthic microeukaryotes in intertidal zones, with homogenizing dispersal making a higher contribution to community variation in water than sediment, and heterogeneous selection showing an opposite trend (Figure 5B). These findings indicate that stochastic processes have a large impact on both planktonic and benthic microeukaryote communities in intertidal zones. This may be attributed to the high and random dispersal of microeukaryotes in water compared to their more restricted dispersal in sediments. Nevertheless, daily tidal fluctuations, surface winds, and strong mixing between water and surface sediments could enhance the dispersal ability of benthic microeukaryotes in intertidal zones. Chen et al. (2017) reported similar results in their investigation of microeukaryotes in sandy sediments and coastal waters of Xiamen Bay. It was concluded that microeukaryotes fitted well to a Neutral Community Model (NCM), suggesting a strong effect of stochastic process on microeukaryotes in intertidal and coastal zones (Chen et al., 2017). The findings of the present study suggest that tide- and wind-induced mixing might generate a relatively more homogeneous environment in the water compared to the sediment, and this is likely the reason why homogenizing dispersal contributed more to the former. Also, sediment showed a significantly higher environmental heterogeneity than that of water (Supplementary Figure S2); this could be the reason why heterogeneous selection imposed a relatively larger effect on microeukaryotes in sediment compared to water.

In the present study, we found that planktonic and benthic microeukaryotes harbored distinct alpha- and beta-diversity in intertidal zones, which is consistent with the diversity distribution patterns of both communities in coastal environments. Organism size and seasonal variation had a significant impact on the community structure of microeukaryotes in intertidal zones, whereas these two factors had much less effect on the alpha diversity of planktonic and benthic microeukaryotes. Intertidal microeukaryote communities in water and sediment were mainly structured by stochastic ecological processes, despite distinct differences in the community composition of planktonic and benthic microeukaryotes. The absence of replicates for each sampling site makes it impossible to evaluate the variation within each site. Besides, intertidal microeukaryotes were only sampled in spring and summer. Therefore, future studies should include sample replicates and be collected during all four seasons in order to validate the observed patterns in the present study.

## Data Availability Statement

The datasets generated for this study can be found in the NCBI Sequence Read Archive SRP201828.

## Author Contributions

PS designed the experiments. JK and YW performed the experiments. PS, JK, and YW analyzed the data. All authors wrote the paper.

### Conflict of Interest

The authors declare that the research was conducted in the absence of any commercial or financial relationships that could be construed as a potential conflict of interest.
