# Supplementary material for: Diversity Distribution and Assembly Mechanisms of Planktonic and Benthic Microeukaryote Communities in Intertidal Zones of Southeast Fujian, China
Source: Front Microbiol. 2019 Nov 15;10:2640. doi: 10.3389/fmicb.2019.02640 (PMC6872677; doi:10.3389/fmicb.2019.02640)

Supplementary Table S1 | Sampling locations and dates and types of samples collected in intertidal zones of southeast Fujian, China. AT, Ao Tou; AY, Ao Yuan; DD, Da Deng; ML, Ma Luan; QL, Qiong Lin; QT, Qiao Tou; QTC, Qiong Tou Cun; SH, Shen Hu; SY, Song Yu; WT, Wei Tou; XS, Xiang Shan; YW, Yan Wu; ZC, Zhang Cuo. Date, Month/Day/Year; Mi, Micro-sized planktonic sample; Na, Nano-sized planktonic sample; Be, benthic sample.

**Sites**	**Latitude (N)**	**Longitude (E)**	**Spring**		**Summer**	
			**Date**	**Types**	**Date**	**Types**
AT	24.54°	118.23°	02/27/2016	Mi/Na	-	-
AY	24.57°	118.10°	02/26/2016	Mi/Na/Be	08/23/2016	Be
DD	24.55°	118.32°	02/27/2016	Na	-	-
ML	24.55°	118.04°	02/26/2016	Mi/Na/Be	08/23/2016	Be
QL	24.46°	118.37°	02/27/2016	Mi/Na	-	-
QT	24.61°	118.42°	02/27/2016	Na	-	-
QTC	24.63°	118.18°	02/27/2016	Be	08/24/2016	Be
SH	24.63°	118.67°	02/28/2016	Mi/Be	08/25/2016	Be
SY	24.46°	118.04°	02/26/2016	Mi/Na/Be	08/23/2016	Be
WT	24.52°	118.57°	02/28/2016	Mi/Na/Be	08/25/2016	Be
XS	24.49°	118.20°	03/05/2016	Mi/Na/Be	-	-
YW	24.43°	118.09°	03/05/2016	Mi/Na	-	-
ZC	24.62°	118.50°	02/28/2016	Mi/Na/Be	08/25/2016	Be

Supplementary Table S2 | Spearman rank correlation tests for the correlations between environmental variables and alpha diversity indices of planktonic microeukaryotes. *P-values* are corrected using the “holm” method. Significant *P*-values (< 0.05) are in bold. All represents all 21 planktonic samples. Microplankton (10 samples) and Nanoplankton (11 samples) refer to size-fractionated water samples collected in spring. PD, phylogenetic diversity; As, arsenic; Cd, cadmium; Co, cobalt; Cu, copper; Fe, iron; Mn, manganese; Ni, nickel; Se, Selenium; Zn, zinc.

**Variables**	**All**						**Microplankton**						**Nanoplankton**					
	**Richness**		**PD**		**Shannon**		**Richness**		**PD**		**Shannon**		**Richness**		**PD**		**Shannon**	
	**r**	***P***	**r**	***P***	**r**	***P***	**r**	***P***	**r**	***P***	**r**	***P***	**r**	***P***	**r**	***P***	**r**	***P***
**Latitude**	-0.388	0.101	-0.271	0.262	0.02	0.934	-	0.332	-0.2	0.606	-0.683	0.042	-0.442	0.2	-0.345	0.328	0.588	0.074
**Longitude**	-0.115	0.638	-0.123	0.615	0.027	0.912	0.167	0.668	0.217	0.576	-0.15	0.7	-0.503	0.138	-0.455	0.187	0.224	0.533
**pH**	0.072	0.771	0.171	0.483	0.024	0.923	0.167	0.667	0.192	0.62	0.452	0.222	-0.116	0.751	0.158	0.663	-0.176	0.626
**Salinity**	0.022	0.929	0.092	0.709	-0.027	0.912	0.133	0.732	0.217	0.576	-0.117	0.765	-0.176	0.627	0.018	0.96	0.042	0.907
**As**	0.276	0.254	0.389	0.1	-0.182	0.455	0.333	0.381	0.617	0.077	-0.033	0.932	0.248	0.489	0.285	0.425	-0.152	0.676
**Cd**	-0.013	0.957	-0.005	0.983	0.198	0.416	-0.3	0.433	-0.517	0.154	-0.05	0.898	0.236	0.511	0.418	0.229	0.406	0.244
**Co**	-0.018	0.94	-0.092	0.709	-0.439	0.06	-	0.46	-0.4	0.286	-0.417	0.265	0.321	0.365	0.152	0.676	-0.527	0.117
**Cu**	-0.122	0.618	-0.137	0.575	0.143	0.558	0.083	0.831	0.133	0.732	-0.383	0.308	-0.188	0.603	-0.248	0.489	0.491	0.15
**Fe**	-0.084	0.734	-0.114	0.641	-0.133	0.587	0.233	0.546	0.383	0.308	-0.1	0.798	-0.37	0.293	-0.491	0.15	-0.139	0.701
**Mn**	-0.105	0.67	-0.113	0.646	-0.064	0.794	-0.3	0.433	-0.233	0.546	**-0.7**	**0.036**	0.091	0.803	-0.067	0.855	0.285	0.425
**Ni**	-0.268	0.266	-0.271	0.262	-0.276	0.254	**-0.7**	**0.036**	**-0.733**	**0.025**	-0.467	0.205	0.127	0.726	0.006	0.987	-0.176	0.627
**Se**	-0.143	0.558	-0.155	0.527	-0.172	0.482	0.267	0.488	0.45	0.224	0.133	0.732	-0.539	0.108	**-0.636**	**0.048**	-0.358	0.31
**Zn**	-0.011	0.963	-0.215	0.377	0.07	0.777	0.033	0.932	-0.083	0.831	-0.217	0.576	0.055	0.881	-0.297	0.405	0.139	0.701

Supplementary Table S3 | Spearman rank correlation tests for the correlations between environmental variables and alpha diversity index of benthic microeukaryotes. *P-values* are corrected using the “holm” method. Significant *P*-values (< 0.05) are in bold. All represents all 15 benthic samples. Spring (8 samples) and Summer (7 samples) refer to sediment samples collected in spring and summer, respectively. PD, phylogenetic diversity; As, arsenic; Cd, cadmium; Cr, chrome; Cu, copper; Ni, nickel; Pb, lead; Zn, zinc; NOx, nitrate, and nitrite; PO4, dissolved reactive phosphorus; Si, dissolved silicate; Water*%,* water content of the sediment. *≤0.001, 0.001-0.002*, *0.004-0.008*, *0.008-0.016, 0.016-0.032*, *0.032-0.063*, *0.063-0.125*, *0.125-0.25*, *0.25-0.5* and *0.5-1* are different grain size ranges of sediments in unit of millimeter.

Variables	All						Spring						Summer					
	Richness		PD		Shannon		Richness		PD		Shannon		Richness		PD		Shannon	
	r	*P*	r	*P*	r	*P*	r	*P*	r	*P*	r	*P*	r	*P*	r	*P*	r	*P*
**Latitude**	-0.063	0.824	-0.397	0.143	-0.178	0.526	-0.31	0.456	-0.476	0.233	-0.429	0.289	0.071	0.879	-0.321	0.482	0.107	0.819
**Longitud**	-0.185	0.509	-0.203	0.468	0.023	0.934	-0.357	0.385	-0.667	0.071	-0.048	0.911	0.036	0.939	0.036	0.939	0.179	0.702
**pH**	-0.021	0.939	-0.036	0.899	0.048	0.864	0.707	0.05	0.323	0.435	0.096	0.821	**-0.955**	**0.001**	-0.36	0.427	**-0.847**	**0.016**
**Salinity**	-0.35	0.201	-0.117	0.679	0.104	0.712	-0.61	0.108	**-0.903**	**0.002**	-0.244	0.56	0.198	0.67	0.631	0.129	0.414	0.355
**As**	-0.068	0.81	-0.068	0.81	-0.289	0.296	0.524	0.183	0.286	0.493	0.19	0.651	-0.464	0.294	-0.321	0.482	-0.679	0.094
**Cd**	-0.496	0.06	-0.354	0.196	**-0.654**	**0.008**	0	1	0.333	0.42	-0.429	0.289	**-0.857**	**0.014**	**-0.821**	**0.023**	**-0.964**	**0**
**Cr**	-0.096	0.732	-0.154	0.585	-0.161	0.567	0.143	0.736	0.238	0.57	0.238	0.57	-0.571	0.18	-0.393	0.383	-0.571	0.18
**Cu**	-0.275	0.321	-0.307	0.265	**-0.525**	**0.044**	0.286	0.493	0.238	0.57	-0.333	0.42	**-0.857**	**0.014**	-0.679	0.094	**-0.857**	**0.014**
**Ni**	-0.168	0.55	-0.018	0.95	0.025	0.93	0.19	0.651	0.333	0.42	0.524	0.183	-0.429	0.337	-0.214	0.645	-0.393	0.383
**Pb**	0.018	0.95	0.132	0.639	-0.361	0.187	0.19	0.651	**0.738**	**0.037**	-0.071	0.867	-0.5	0.253	-0.393	0.383	-0.643	0.119
**Zn**	-0.154	0.585	-0.161	0.567	-0.175	0.533	0.405	0.32	0.381	0.352	-0.024	0.955	**-0.857**	**0.014**	-0.679	0.094	**-0.857**	**0.014**
**NO_X**	0.443	0.098	0.286	0.302	**0.521**	**0.046**	0.643	0.086	0.048	0.911	0.381	0.352	0.5	0.253	0.714	0.071	0.75	0.052
**PO4**	0.368	0.177	-0.004	0.99	-0.029	0.919	0.19	0.651	-0.357	0.385	-0.548	0.16	0.643	0.119	0.357	0.432	**0.786**	**0.036**
**Si**	-0.021	0.94	0.086	0.761	-0.254	0.362	-0.167	0.693	0.143	0.736	-0.381	0.352	0.214	0.645	-0.036	0.939	-0.143	0.76
**Water%**	-0.504	0.056	-0.168	0.55	-0.011	0.97	-0.286	0.493	0.214	0.61	0.381	0.352	**-0.857**	**0.014**	-0.464	0.294	**-0.821**	**0.023**
**Bacteria**	-0.189	0.499	-0.389	0.152	0.104	0.713	0.024	0.955	-0.143	0.736	0.048	0.911	-0.036	0.939	-0.357	0.432	-0.107	0.819
**Chl*a***	-0.279	0.315	-0.464	0.081	-0.379	0.164	-0.429	0.289	-0.19	0.651	-0.19	0.651	-0.179	0.702	**-0.786**	**0.036**	-0.429	0.337
**≤0.001**	-0.418	0.121	-0.075	0.791	-0.164	0.558	-0.571	0.139	-0.31	0.456	-0.333	0.42	-0.286	0.535	0.357	0.432	-0.214	0.645
**0.001-**	**-0.621**	**0.013**	-0.346	0.206	-0.261	0.348	-0.619	0.102	-0.286	0.493	-0.095	0.823	-0.679	0.094	-0.214	0.645	-0.714	0.071
**0.002-**	**-0.582**	**0.023**	-0.318	0.248	-0.279	0.315	-0.619	0.102	-0.286	0.493	-0.095	0.823	-0.643	0.119	-0.357	0.432	**-0.786**	**0.036**
**0.004-**	**-0.536**	**0.04**	-0.229	0.413	-0.239	0.39	-0.524	0.183	-0.143	0.736	0.024	0.955	-0.679	0.094	-0.214	0.645	-0.714	0.071
**0.008-**	-0.364	0.182	-0.071	0.8	-0.164	0.558	-0.357	0.385	0.024	0.955	0.048	0.911	-0.607	0.148	-0.107	0.819	-0.571	0.18

**0.016-**	0.007	0.98	0.286	0.302	-0.025	0.93	-0.143	0.736	0.429	0.289	0.095	0.823	-0.179	0.702	0.214	0.645	-0.071	0.879
**0.032-**	0.221	0.428	0.371	0.173	0.061	0.83	0.595	0.12	**0.762**	**0.028**	0.071	0.867	-0.071	0.879	0.321	0.482	0.214	0.645
**0.063-**	0.35	0.201	0.221	0.428	0.236	0.398	0.5	0.207	0.024	0.955	-0.143	0.736	0.286	0.535	0.5	0.253	0.571	0.18
**0.125-0.25**	0.25	0.369	-0.025	0.93	0.164	0.558	0.19	0.651	-0.357	0.385	-0.19	0.651	0.643	0.119	0.071	0.879	0.536	0.215
**0.25-0.5**	0.261	0.348	-0.004	0.99	0.046	0.869	0.238	0.57	0.024	0.955	-0.167	0.693	0.714	0.071	0	1	0.607	0.148
**0.5-1**	0.148	0.599	0.038	0.893	-0.023	0.934	-0.252	0.548	-0.096	0.821	-0.419	0.301	0.595	0.159	-0.072	0.878	0.487	0.268

Supplementary Figure S1 | Principal component analysis of environmental factors measured for both seawater (11) and sediment (15) samples in intertidal zones. Environmental factors were standardized to zero mean and unit variance and used to calculate the Euclidean distance of all samples. Salinity, pH, the concentration of Cu, Zn, Ni, Cd, As were included in the principal component analysis. AT, Ao Tou; AY, Ao Yuan; DD, Da Deng; ML, Ma Luan; QL, Qiong Lin; QT, Qiao Tou; QTC, Qiong Tou Cun; SH, Shen Hu; SY, Song Yu; WT, Wei Tou; XS, Xiang Shan; YW, Yan Wu; ZC, Zhang Cuo.

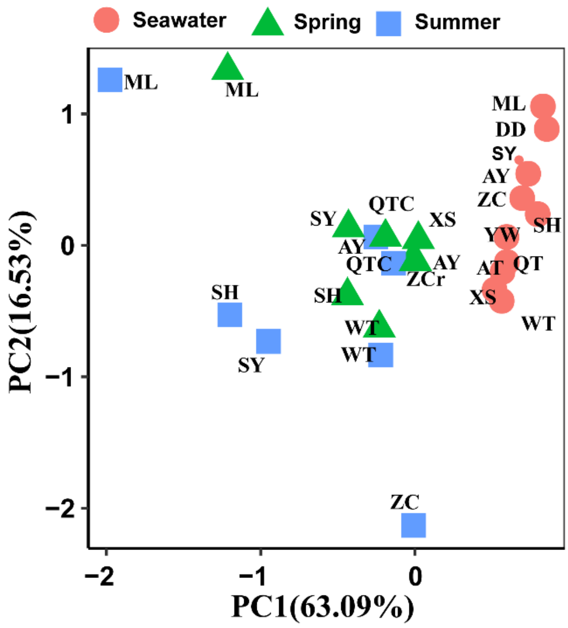

Supplementary Figure S2 | Comparing shared environmental variables measured for both water (11) and sediment (15) samples in intertidal zones **(A)**, Salinity **(B)**, pH **(C)**, As **(D)**, Cd **(E)**, Cu **(F)**, Ni **(G)**, Zn **(H)**. Significance was tested by t-test.

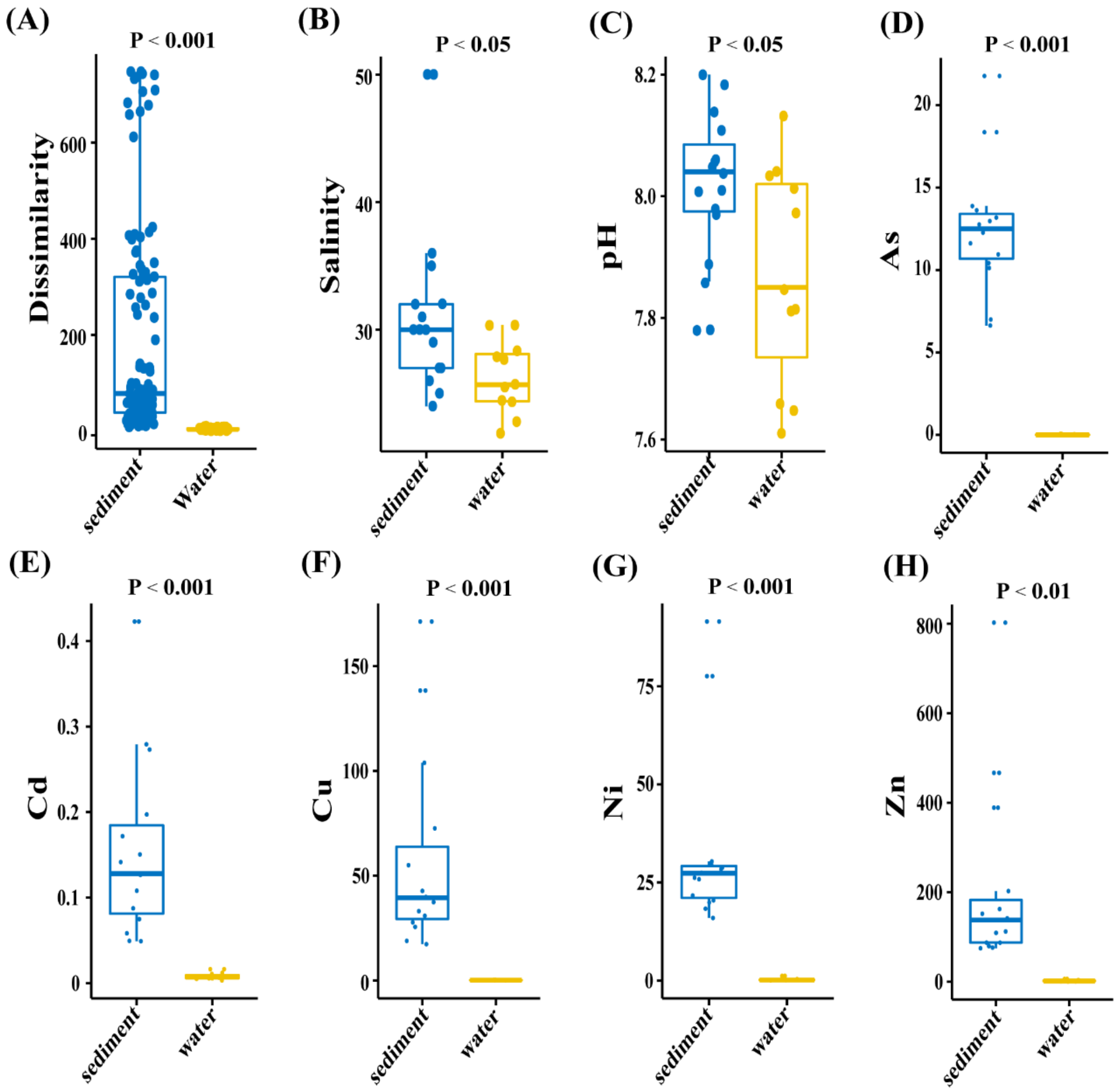

Supplementary Figure S3 | Heatmap showing the distribution and composition of differential OTUs across plankton and benthos (A), Microplankton (10) and Nanoplankton (11) (B), Spring (8) and Summer (7) (C) samples. Log-transformed relative sequence abundance of OTUs were identified using edgeR and presented in mean value of different groups. Only OTUs with sequences making up more than 0.5% of total sequences were used. OTUs in bold are the the ones enriched in benthos/Microplankton/Spirng samples.

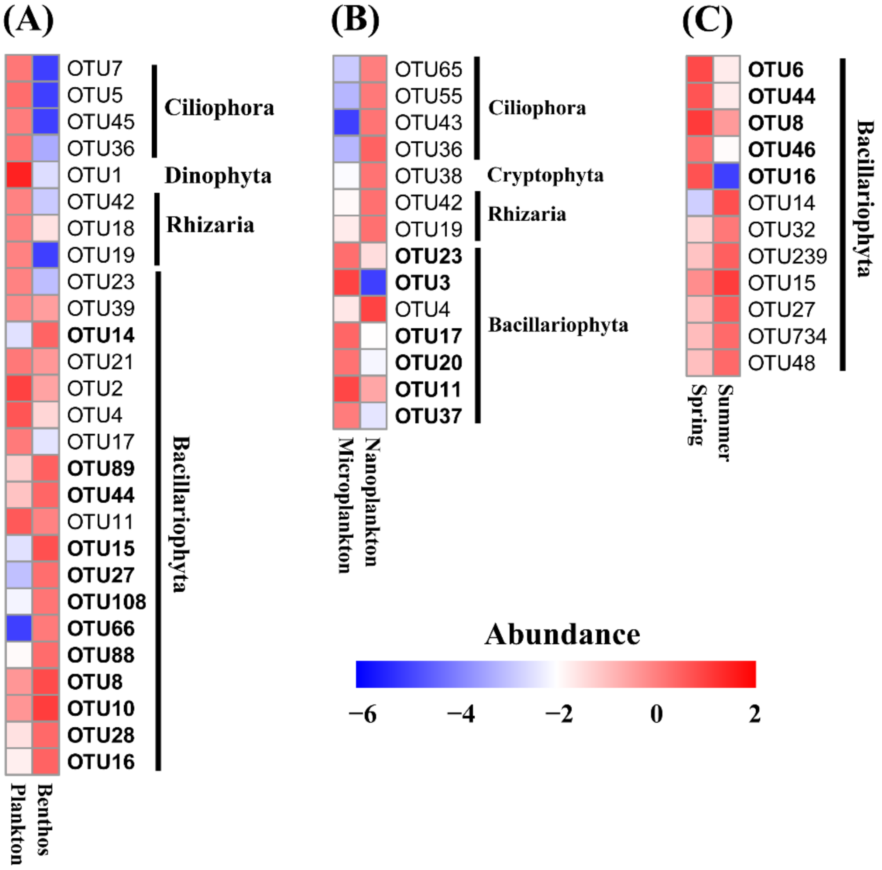

Supplementary Figure S4 | Spearman's rank correlations between the pairwise geographic distance of sampling sites and community dissimilarity of intertidal microeukaryotes based on Bray-Curtis distances.

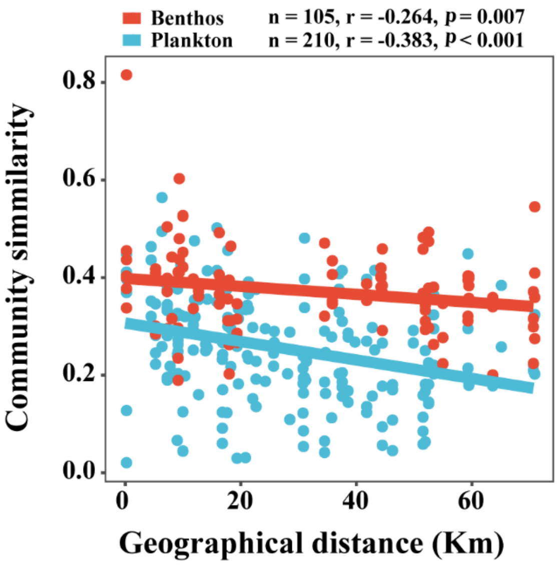

Supplementary Figure S5 | Venn diagram showing unique and shared β-diversity of planktonic and benthic intertidal microeukaryotes based on OTU abundance. Plankton (21 samples) includes Microplankton and Nanoplankton, while Benthos (15 samples) includes samples collected in both Spring and Summer.

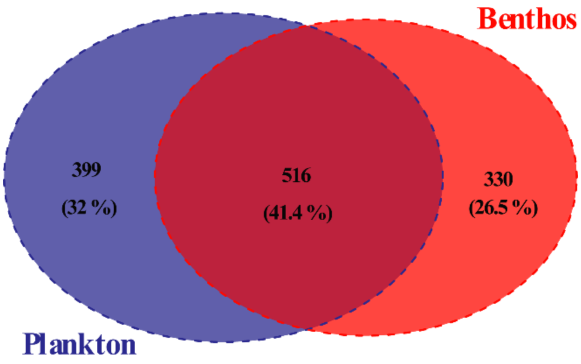

Supplement: Supplementary file 1 [file Data_Sheet_1.PDF]
